# Promising Probiotic Candidates for Sustainable Aquaculture: An Updated Review

**DOI:** 10.3390/ani14243644

**Published:** 2024-12-17

**Authors:** Seyed Hossein Hoseinifar, Mehwish Faheem, Iram Liaqat, Hien Van Doan, Koushik Ghosh, Einar Ringø

**Affiliations:** 1 Department of Fisheries, Gorgan University of Agricultural Sciences and Natural Resources, Gorgan 4913815739, Iran; 2 Department of Zoology, GC University Lahore, Punjab 54000, Pakistan; m.faheem@missouri.edu (M.F.); iramliaq@hotmail.com (I.L.); 3 Department of Animal and Aquatic Sciences, Faculty of Agriculture, Chiang Mai University, Chiang Mai 50200, Thailand; hien.d@cmu.ac.th; 4 Functional Feed Innovation Center (FuncFeed), Faculty of Agriculture, Chiang Mai University, Chiang Mai 50200, Thailand; 5 Aquaculture Laboratory, Department of Zoology, The University of Burdwan, Burdwan 713104, West Bengal, India; kghosh@zoo.buruniv.ac.in; 6 Faculty of Bioscience, Fisheries and Economics, Norwegian College of Fishery Science, UiT The Arctic University of Norway, 9019 Tromsø, Norway

**Keywords:** probiotics, fish, shellfish, growth, immune response, disease resistance

## Abstract

With the intensification of aquaculture to meet the rising demands of fish and shellfish, disease outbreaks during the larval and adult stages are a major challenge faced by aqua culturists. As the prophylactic use of vaccines and antibiotics has several limitations, research is now focused on sustainable alternatives to vaccines and antibiotics, e.g., medicinal plants, probiotics, postbiotics, prebiotics, and synbiotics, as promising candidates to strengthen the immune response of fish and shellfish and to control disease outbreaks. With respect to probiotics, numerous studies are available revealing their health-promoting and beneficial impacts in aquaculture. However, most studies focus on *Bacillus* and *Lactobacillus* species. Keeping in view the positive effects of probiotic lactic acid bacteria in aquaculture, researchers are now looking for other probiotic bacteria that can be used in aquaculture. Recently, many non-lactic acid bacteria (non-LAB), which are mainly host-associated, have been reported to reveal beneficial effects in fish and shellfish aquaculture. The main non-LAB probiotic genera are *Bifidobacterium*, *Clostridium*, *Microbacterium*, *Micrococcus*, *Paenibacillus*, *Acinetobacter*, *Alcaligenes*, *Enterobacter*, *Phaeobacter Pseudoalteromonas*, *Pseudomonas*, *Pseudomonas*, and *Vibrio*. Despite the promising effects of non-LAB probiotics, comparably, there is limited available information in this context. This review focuses only on probiotic strains that are non-LAB, mostly isolated from the host digestive tract or rearing water, and discusses their beneficial effects in fish and shellfish aquaculture. This review will provide detailed information on the use of various non-LAB bacteria and provide a roadmap to future studies on new probiotics for sustainable aquaculture.

## Simple Summary

Aquaculture suffers from losses due to disease outbreaks, and to keep the industry sustainable, there are strict limitations on antibiotic use. Therefore, several alternatives have been suggested, such as probiotics. *Bacillus* and *Lactobacillus* species are among the most studied and advised probiotics for aquaculture. However, researchers are now looking for other probiotic bacteria that can be used in aquaculture. In this context, non-lactic acid bacteria (non-LAB), which are mainly host-associated, should have promising effects on fish and shellfish. Given the potential and advantages of this kind of probiotics, the present review paper provides detailed information on the use of various non-LAB bacteria and provides a roadmap to future studies on new probiotics for sustainable aquaculture.

## 1. Introduction

Animal-based protein is a primary requirement for the growing human population. Wild catches of fish and shellfish provide premium-quality protein, but a marked decline in wild catches has been reported over the past two decades. Now, the aquaculture industry is mainly responsible for the supply of fish and shellfish. Protein from fish and shellfish farming provides a promising sustainable solution to cope with the ever-growing protein demand and solve the problem of malnutrition. 

Even though the aquaculture industry is flourishing rapidly, many factors are impeding the progress of this industry. The occurrence of disease and high larval mortality during early rearing are significant challenges faced by the aquaculture industry. The mass mortality of fish larvae and adult fish results in substantial economic loss. Various factors contribute to high mortality; poor water quality and management of the rearing system, the unavailability of live food such as zooplankton and phytoplankton, nutrition deficiency in larval feed, and the presence of pathogenic bacteria are major contributing factors [1]. 

The presence of opportunistic and pathogenic bacteria in the culture system is a significant challenge that can eradicate a complete batch of fish, resulting in the loss of billions of dollars. Antibiotics have been used to control the spread of infection caused by such bacteria, but the prolonged use of antibiotics results in antibiotic resistance [2]. Moreover, the presence of antibiotic residue in fish and water can pose threats to humans and is a concern for food security. Vaccines have also been used for the prevention of infection in fin fish. Still, the use of vaccines in shellfish culture is of little interest because, in contrast to vertebrates, invertebrates lack the cellular machinery and immunoglobulins needed to mount a response against pathogens. They solely rely on their innate immune system, which, until recently, was not considered a target for vaccines [3].

The word probiotic was first used by Lilly and Stillwell [4]. Since then, numerous studies have focused on the bio-control of pathogenic bacteria in aquaculture using probiotics and other biologically derived products, like paraprobiotics and prebiotics, and researchers have tried to explore economical, eco-friendly, and sustainable alternatives to antibiotics. The use of bacterial species is a very promising approach to limiting the proliferation of opportunistic and pathogenic bacteria that are present in fish rearing systems and feed [5]. 

Several bacterial species have been used as probiotics in aquaculture [6], but most attention has been focused on lactic acid bacteria (LAB) [7,8,9,10], bacilli (e.g., [11,12,13,14,15]), and yeasts [16]. Therefore, the current review presents an updated overview of the several other bacteria species proposed as interesting probiotics to improve fish and shellfish health. As single-strain probiotics may not be effective in all culture systems and a given probiotic may affect different fish species differently, questions remain regarding the use of host-associated microbes and the use of other bacterial species as probiotics in aquaculture. Therefore, research attempts regarding the administration of probiotics other than LAB and non-LAB in aquaculture have gained interest, as these species are mainly host-associated probiotics (HAP), which may be more effective than probiotics from other sources. The advantages of HAP were discussed in detail in our previous review paper [17]. Given the importance of probiotics other than LAB and non-LAB probiotics for sustainable aquaculture, the present review paper tries to provide a comprehensive overview of these probiotics. Considering the point that two recently published review papers by Ringø et al. [18] and Rohani et al. [19] presented some information on these probiotics, studies discussed in the abovementioned reviews are not presented in the text and tables of this paper to avoid duplication. 

## 2. Probiotics and Their Possible Mode of Action

Probiotics are “*live microorganisms that*, *when provided in sufficient proportions*, *impart a health benefit on the host*”. The primary mechanism through which probiotics exert their action is direct and indirect antagonism. Probiotics inhibit the growth of harmful opportunist bacteria by competing for resources like iron, nutrients, and attachment sites [20], producing bacteriocins [21]. Probiotic administrations to fish and shellfish not only compete with harmful bacteria, but also improve growth, digestive enzyme activity, and gut morphology, strengthening the immune system and disease resistance towards pathogens (e.g., Nayak [22]; Hoseinifar, Sun, Wang, and Zhou [6]; Ringø, Hoseinifar, Ghosh, Doan, Beck, and Song [8]; Ringø, Li, Doan, and Ghosh [18]; Soltani, Ghosh, Hoseinifar, Kumar, Lymbery, Roy, and Ringø [11]; Soltani, Abu-Elala, and Ringø [13]; Ghosh, Harikrishnan, Mukhopadhyay, and Ringø [16]; Van Doan [12]). The administration of probiotics to fish stimulates the innate immune system and results in increased lysozyme and complement activity, accompanied by an increased production of pro-inflammatory cytokines [22]. This produces metabolites such as hydrolytic coenzymes that have the potential to improve the digestive proteolytic and amylolytic activity of the host, resulting in improved growth. 

## 3. Application of Promising Probiotics in Fish

### 3.1. Gram-Positives

In a recent review, Ringø, Li, Doan, and Ghosh [18] presented some information on probiotic administration in fish. To avoid duplication, these studies are not presented in the text of this study or Table 1.

### 3.2. Bifidobacterium

In a study on Japanese sea bass (*Lateolabrax japonicus*), *B. animalis* subsp. *lactis* (10^10^ CFU g^−1^) was administered for one month and increased growth, survival, serum antioxidant capacity and innate immunity; it modulated hindgut microbiota but decreased levels of oxidants (H_2_O_2_ and malondialdehyde) [23]. The authors showed that Firmicutes and Proteobacteria were the major bacterial phyla in the intestinal microbiota of juvenile Japanese seabass. Feeding on *B. animalis* subsp. *lactis* significantly increased the relative abundance of Patescibacteria and decreased Fusobacteria. 

Yisa, Ibrahim, Tsadu, and Yakubu [24] conducted a 56-day feeding trial with hybrid catfish (*Heteroclarias*) fingerlings to determine the effect of multi-strain probiotics (MSPs) consisting of *B. bifidum* and *Lactobacillus acidophilus*, and the authors revealed improved growth performance and survival, affected immune response, and modulated gut microbiota. However, the study of the gut microbiota was preliminary, and the authors just checked the strains based on Gram-staining. Based on these tests, the authors claimed that the presence of Gram-positive, rod-shaped bacteria in Heteroclariasfed probiotics led to the establishment of normal gut microbiota.

Ayyat, Labib, and Mahmoud [25] illustrated the individual (10^7^ cells/100 g diet) as well as combined effects of dietary *B. bifidum* on the growth profile and disease resistance of Nile tilapia (*Oreochromis niloticus*) fingerlings against *Aeromonas hydrophila* infection. The results showed that fish fed on supplemented diets displayed increased growth rates, feed consumption, and survival rates. Jha et al. [91] reported the effects of dietary *B. bifidum* supplementation on the growth of rohu (*Labeo rohita*) hatchlings and fry in outdoor tanks for 30 days. The results presented that the rohu hatchlings and fry fed the probiotic-supplemented diets displayed improved growth and survival rates compared to unsupplemented ones.

In a three-month study on African sharp-tooth catfish (*Clarias gariepinus*), Ayoola et al. [92] revealed that varying levels of dietary *Bifidobacterium* supplementation improved weight gain, specific growth rate, and protein efficiency ratio. 

Dima, Sîrbu, Patriche, Cristea, Coadă, and Plăcintă [27] investigated the effect of MSPs, bifidobacteria, and lactobacilli on common carp (*Cyprinus carpio*). The authors revealed a positive effect on growth performance and an increase in the number of erythrocytes, haemoglobin synthesis, and lymphocytes. 

The administration of MSPs to Nile tilapia in a 90-day study by Silva, Salomão, Mareco, Dal Pai, and Santos [28] showed a positive effect on growth, muscle growth, gene expression (MyoG, MyoD, IGF-1, and myostatin genes), and the number of intestinal villi. 

Sayed Hassani, Jourdehi, Zelti, Masouleh, and Lakani [29] illustrated the effects of dietary *B. bifidum* on the growth and immune status of Siberian sturgeon (*Acipenser baerii*) over 56 days. The results displayed that fish fed *B. bifidum*-complemented diets showed increased specific growth rate and percentage weight gain, and serum IgM and lysozyme activity increased in fingerlings. 

Bhujel, Jha, and Anal [33] revealed the improved survival and specific growth rate of rohu hatchlings and advanced fry fed MSPs consisting of *Bifidobacterium*, *Lactobacillus*, *Saccharomyces cerevisiae*, *Spirulina*, and phytase. Based on their results, the authors concluded that beneficial effects of the multi-strain administration were found in hatchlings and fry, but not in advanced fry.

Cavalcante, Telli, Tachibana, de Carla Dias, Oshiro, Natori, da Silva, and Ranzani-Paiva [34] evaluated the effects of dietary *Bifidobacterium* species supplementation on the growth and disease resistance of Nile tilapia against *A. hydrophila* in a 63-day study. They showed that fish fed probiotic diets showed improved growth performance and survival rates when challenged with *A. hydrophila* infection. Furthermore, fish fed the supplemented diets showed improved gut health, as well as increased lysozyme activity, relative protection level, and phagocytic capacity and indices.

In a study that administered *B. longum*, *B. thermophilum*, and *Lactobacillus acidophilus* to Nile tilapia fingerlings for three months, Khalafalla, Ibrahim, Zayed, Awad, and Mohamed [32] evaluated five diets with different amounts of bacteria per kilogram: 0, 1.0, 2.0, 3.0, and 4.0 g. The findings showed that fish fed a diet supplemented with 4.0 g of bacterial combination had the highest final body weight, weight gain, specific growth rate, feed intake, and the best feed conversion ratio.

### 3.3. Brevibacillus

In an early study, Mahdhi, Kamoun, Messina, Santulli, and Bakhrouf [35] reported that a *Brevibacillus brevis* strain inhibited the in vitro growth of fish pathogenic bacteria. *Artemia* was used as a vector for probiotic administration and showed an improved specific growth rate in sea bass (*Dicentrarchus labrax*) larvae. However, as only the growth rate was determined, one cannot conclude that *B. brevis* supplements are suitable for sea bass, as further investigations are needed. In a recent study, Alak, Kotan, Uçar, Parlak, and Atamanalp [82] conducted a feeding trial with rainbow trout to determine the effect of *B. brevis*. They revealed that probiotic administration affected malondialdehyde, as well as significant differences in haematological indices and swimming performance. Yang, Wang, Dai, Liu, Zhang, Zeng, Zeng, Ni, and Pan [37] supplemented crucian carp (*Carassius auratus*) diets with different doses (5 × 10^3^, 5 × 10^4^, and 5 × 10^5^ CFU mL^−1^) of *Brevibacillus laterosporus* for eight weeks, and at the end of the feeding trial found improved water quality, nitrogen concentration, growth performance, and antioxidant capacity in serum, and live and digestive protease and amylase activities were noticed. 

### 3.4. Clostridium

Butyrate-producing bacteria confer health benefits on the host and have been considered candidate probiotics for use in aquaculture. In addition to producing short-chain fatty acids (SCFAs), they secrete amylase and reduce harmful substances like sputum and amines. Clostridium butyricum is frequently added to feed for a variety of animals to inhibit the growth of harmful bacteria in the intestine and encourage the development of *Bifidobacterium* [43]. To avoid overlaps, studies cited in the referenced review are not cited in the current review. 

Li et al. [93] reported the effects of dietary *C. autoethanogenum* supplementation on the growth rate and immunity of Jian carp (*Cyprinus carpio*) juveniles. The results showed that fish fed supplemented diets demonstrated improved percentage weight gain and specific growth rate. Furthermore, the mRNA expression of growth-related genes (PepT-1, PepT-2, and IGF-1) and TOR signalling pathway-related genes (TOR, 4E-BP2, and S6K1) was improved by the dietary *C. autoethanogenum* supplementation.

The administration of *C. autoethanogenum* (CAP) protein to largemouth bass (*Micropterus salmoides*) by Li, Wang, Zhang, and Wang [38] revealed improved growth performance, antioxidant capacity, and intestinal short fatty acids (SCFAs). Intestinal inflammatory response and permeability decreased with CAP administration, and the relative abundance of opportunistic pathogens, including *Ralstonia*, *Acinetobacter*, *Aeromonas*, and Proteobacteria, in intestinal content was attenuated with CAP.

Chen et al. [94] illustrated the effects of a *C. butyricum (10^8^ CFU g^−1^)-enriched diet* on the growth and gut microbiota of mandarin fish (*Siniperca chuatsi*) for eight weeks. The results showed that perch fed on a *C. butyricum*-enriched diet demonstrated increased weight gain, serum globulins, and lysozyme activity. Additionally, fish fed the enriched diet showed increased healthy gut microbial diversity (*Lactobacillus*, *Bacillus*, and *Paenibacillus*), and *Aeromonas* were reduced. 

Recently, Gao et al. [95] reported the effects *of C. butyricum* on the growth profile, immunity, and digestive enzyme activities of silver pomfret (*Pampus argenteus*) over 60 days. The results indicated that fish fed supplemented diets have enhanced specific growth rates, as well as increased lysozyme activity and IgM concentrations. Furthermore, the activities of digestive enzymes, lipase, amylase, and protease were higher in fish fed *C. butyricum*-supplemented diets compared to unsupplemented ones. Yan et al. (2022) [40] reported that the administration of *C. butyricum* to crucian carp in a 10-day study modulated the gut microbiota and improved the microbial metabolism, while the relative abundance of *Aeromonas* and *Rhodobacter* decreased. 

Zhang, Liang, He, Feng, and Li [41] conducted an 8-week feeding trial with Chinese perch (*Siniperca chuasti*) to determine the effect of *C. butyricum*. The trial revealed decreased growth performance and length of the intestinal villi, which affected nutrient absorption efficiency and hindgut content microbial diversity. The *C. butyricum* group displayed a significantly lower relative abundance in *Xenobiotic* biodegradation and metabolism than the control group. The study of gut microbiota revealed that *C. butyricum* was not able to colonize effectively in the intestinal tract of Chinese perch. Meanwhile, the long-term administration of probiotics reduced the gut microbial diversity and the abundance of some bacteria (*Romboutsia* and *Bacillus*).

In a study using turbot (*Scophthalmus maximus*), *C. butyricum* (10^6^, 10^7^, and 10^8^ CFU g^−1^10^5^ CFU g^−1^) was administered for 56 days [43]. The administration of 10^7^ CFU g^−1^ best improved growth performance, the length of the intestinal villi, and nutrient absorption efficiency. Also, the dietary administration of *C. butyricum* could modulate the intestinal microbiota and increase the relative abundance of potential probiotic-related genera, including *Clostridiales* and *Bacillales*, in turbot. 

Clarifying the effect of C. *butyricum* administration on hybrid groupers (*Epinephelus lanceolatus* ♂ × *E. fuscoguttatus* ♀), Yang, Xun, Huang, Jiang, Huang, Yu, Xu, and Lin [44] found a positive effect on growth performance, intestinal enzymes, chymotrypsin, and α-amylase activity, as well as villus length, muscle thickness, and goblet cells in the intestine. The expression levels of antioxidant-related genes, immune-related genes, and tight junction protein genes in the intestine were also affected. Furthermore, the content of the intestinal microbiota, investigated by 16 S rRNA high-throughput sequencing, showed significant upregulation of the relative abundance of *Mycoplasma*, *Ruminococcus*, *Bacteroides*, *Ruegeria*, *Alistipes*, *Paracoccus*, *Bythopirellula*, *Aquabacterium*, and *Bacillus* and significant downregulation of the relative abundance of *Photobacterium by probiotic administration* compared to the control group.

In addition to direct probiotic administration via feeding, researchers are also working on proteins from microbial sources as replacements for fish meal (FM). In a replacement study by FM, Yu, Wang, Jin, Han, Zhu, Liu, Zhang, Yang, and Xie [39] using two different meals, CAP and protein concentrate at a ratio of 1:1:6:4 (Blend B) showed improved growth and significantly upregulated the mRNA expression of the intestinal proinflammatory cytokines, anti-inflammatory cytokines, and tight junction-related genes (*p* < 0.05). Intestinal tissue morphology was also improved. Based on their results, the authors concluded that Blend B could completely substitute dietary FM and is beneficial to gibel carp growth and health.

Similarly, Yang et al. [96] reported the effects of replacing FM with CAP on the growth performance of largemouth bass (*Micropterus salmoides*) juveniles in an 8-week study. The results showed that fish fed substituted diets showed similar weight gain compared to the control. Also, the fish fed on replaced diets showed increased amylase and protease activities, indicating that CAP could substitute FM in the diet (up to 150 g/kg). 

### 3.5. Microbacterium and Mixtures

In an MSP study by Skjermo, Bakke, Dahle, and Vadstein [45] using *Microbacterium*, *Ruegeria*, *Pseudomonas*, and *Vibrio*, only *Microbacterium* seemed to colonise the Atlantic cod (*Gadus morhua*) larval intestine, even though all candidates were originally isolated from cod larvae intestine. Regarding the modulation of the gut microbiota, the authors concluded that it is difficult to perform sustainable manipulations of the fish larval microbiota by introducing new strains due to the rapid development of the larvae during the larval stages, which continuously changes the selection pressure for the bacteria in the digestive tract. It has been suggested that continuous supply or repeated additions every 3–4 days should be considered.

Lee, Kim, Noh, Lee, Kim, Hasan, Lee, and Jang [46] administered *Microbacterium* spp. and *Rummeliibacillus* sp. to flounder (*Paralichthys olivaceus*) and revealed improved growth performance and a positive effect on the immune parameter myeloperoxidase. Regarding possible modulation of the gut microbiota, the authors noticed a relative abundance of Actinobacteria, Proteobacteria, and Planctomycetes dominant in the probiotic group, in contrast to Firmicutes, Proteobacteria, and Actinobacteria in the intestine of the control flounder.

### 3.6. Micrococcus

In a study on Japanese seabass, Yang, Liu, Jian, Ye, and Sun [47] showed that *Micrococcus aloeverae* and *Micrococcus yunnanensis* stimulated growth performance and survival. However, to fully conclude the effect of the investigated *Micrococcus* species, further studies on the effect on the immune system, gut morphology, gut microbiota, and disease resistance must be evaluated.

### 3.7. Paenibacillus

Lin, Chen, Wen, and Hu [50] conducted an 8-week feeding trial with zebrafish (*Danio rerio*) to determine the effect of *Paenibacillus ehimensis*. They revealed a positive effect on the hepatic mRNA expression of carbohydrate metabolism-related genes and innate immune-related genes, and resistance against *A. hydrophila* and *Streptococcus iniae*. 

In an earlier study, Gupta, Gupta, and Dhawan [51] showed that *Paenibacillus polymyxa* administration to common carp fry was harmless, as neither mortality nor morbidity was observed. In addition, administration stimulated growth performance and lysozyme and respiratory burst activities and resistance towards *A. hydrophila*. 

Yang, Jin, Li, Jiang, Cui, Huang, Rang, Li, and Xia [53] showed in an in vitro test that *P. polymyxa* S3 displayed antagonistic activity against 11 pathogens. Moreover, an in vivo study showed that the bacterium colonises the abdomen; stimulates growth, enzyme activities, acid phosphatase, alkaline phosphatase, glutathione peroxidase, and catalase in serum; up-regulates the expression of antioxidant-related genes, immune-related genes, *Keap1*, *Nrf2*, *C3*, *LZM*, *IgM*, *TLR-4*, and *MyD-88*; and shows resistance against *A. hydrophila*. 

In an MSP study using *Paenibacillus* sp., *B. subtilis*, *Bacillus amyloliquefaciens*, and *Lactobacillus rhamnosus*, Liao, Huang, Lin, Chen, Lee, Lin, Chuang, and Nan [54] showed improved growth performance and resistance against *Vibrio alginolyticus*. 

## 4. Gram-Negatives

Ringø, Li, Doan, and Ghosh [18] presented some information on the probiotic administration of Gram-negatives in fish, and to avoid duplication, these studies are not presented or discussed in the present paper. 

### 4.1. Acinetobacter

Little information is available on the use of *Acinetobacter* as a probiotic in aquaculture [18]. This may be due to reports on opportunistic fish pathogenic agents within the genus. However, a study by Ramírez, Rojas, and Romero [56] presented information on *Acinetobacter* sp. P27 and P33, revealing antimicrobial activity against *Vibrio* 25LT1.

### 4.2. Aeromonas 

Little information is available about *Aeromonas* as a probiotic in fish [18]. A classical study evaluating the probiotic potential of *Aeromonas medium* strain A199 was undertaken by Gibson et al. [97], showing the effects on Pacific oysters (*Crassostrea gigas*) exposed to *Vibrio tubiashii*. In two recent studies, Jinendiran, Archana, Sathishkumar, Kannan, Selvakumar, and Sivakumar [57] and Pacheco, Díaz-Sánchez, Contreras, Villar, Cabezas-Cruz, Gortázar, and de la Fuente [58] revealed a positive effect on common carp and zebrafish administered *Aeromonas veronii* V03 and *A. veronii* with high α-Gal content, respectively.

### 4.3. Alcaligenes

According to Ray and Pattnaik [60], *Alcaligenes faecalis* is often used as a popular biocontrol agent due to its antimicrobial properties. Given its potential nitrogen fixation capability and anaerobic respiration using nitrate or nitrite as a terminal electron acceptor, this genus has been successfully used in the past for biocontrol and biofertilizer. However, in aquaculture, there are limited reports available regarding the administration of different species of *Alcaligenes*. 

In an earlier study, Asaduzzaman, Iehata, Akter, Kader, Ghosh, Khan, and Abol-Munafi [62] isolated *Alcaligenes* sp. AFG22- from the gut microbiota of slow-growing Malaysian mahseer (*Tor tambroides*) to investigate the growth-promoting effect of this host-associated probiotic. Malaysian mahseer were fed with experimental diets inoculated with *Alcaligenes* sp. AFG22 (10^8^ CFU g^−1^) for 90 days, and the results revealed a significant increase in growth performance compared with the control group. Based on the morphometric analysis of muscle, the authors attributed the enhancement in growth to muscle fibre hypertrophy [63]. In accordance with this finding, the transcriptomic analysis revealed the significant upregulation of GH and IGF-1 in the probiotic-fed group. 

Later, the effects of *Alcaligenes faecalis* Y311 on water quality, some physiological parameters, and the gut microbiota of the Nile tilapia were investigated by Wang, Yi, Lu, Gao, Liu, Huang, Li, and Zhu [61]. The probiotic strain was previously isolated from culture tank sediments. The duration of the trial was three months, and during this period, *A. faecalis* was added to the culture water at a rate of 10^4^ CFU/mL. The results revealed that the administration of the probiotic could significantly improve water quality by decreasing total phosphorus. Probiotic treatment also significantly increased the alkaline phosphatase activities in the intestine and the gill. Also, the study of gut microbiota revealed that the treatment of the water with *A. faecalis* showed a beneficial effect by increasing beneficial bacteria such as *Cetobacterium*, *Methylobacterium*, and *Tepidimonas*. Based on these findings, the authors claimed the potential of *A. faecalis* to improve mucosal immunity. 

Overall, although limited studies are available on the use of *A. faecalis* in aquaculture, these findings show that the strain could be considered a promising probiotic.

### 4.4. Enterobacter

*Enterobacter* belongs to the family of Enterobacteriaceae; it is a Gram-negative, rod-shaped bacteria and is commonly isolated from soil, water, and animals’ gut microbiota [66]. To the best of our knowledge, limited studies are available regarding the potential application of Enterobacter as a probiotic in aquaculture. However, the available literature clearly shows its potential due to (1) being safe to host and (2) revealing antibacterial activity against a wide range of pathogens, such as Aeromonas, Pseudomonas, etc. [98]. 

Recently, the effects of *Enterobacter asburiae* E7 as a probiotic for common carp aquaculture were studied by Li, Zhang, Wu, Qu, Wang, Wei, Li, and Ling [66]. The authors first isolated *E. asuriae* from the gut microbiota of carp and checked its bactericidal activity against a wide range of pathogens. The strain passed standard probiotic screening tests and then was added to the carp diet for four weeks at a rate of 10^7^ CFU/g. Although feeding on the probiotic diet had no significant effect on growth performance, it up-regulated immune gene expression, and these findings were subsequently approved in the challenge test with *Aeromonas veronii*, revealing the significantly higher resistance of probiotic-fed carp compared to the controls. The authors concluded that the strain could be used as a promising immunostimulant for sustainable carp aquaculture.

### 4.5. Phaeobacter

*Phaeobacter* belongs to the Roseobacter group and is mainly reported in marine aquaculture systems. According to Roager, Athena-Vasileiadi, Gram, and Sonnenschein [71], it is a safe and promising probiotic for aquaculture. Makridis, Kokou, Bournakas, Papandroulakis, and Sarropoulou [68] reported that *Phaeobacter* isolated from the yolk sac of Atlantic bonito (*Sarda sarda*) larvae showed resistance against the pathogenic bacteria *Vibrio anguillarum* in vitro. When administered to European seabass (*Dicentrarchus labrax*), the larvae improved specific growth rates and resistance against *Vibrio harveyi*. Also, a study of the gut microbiota via denaturing gradient gel electrophoresis (DGGE) revealed an increase in microbial community richness. However, these beneficial effects were not reported after probiotic supplementation stopped. In studies illustrating host–microbiome interactions, *Phaeobacter* was shown to modulate host gene expressions, thereby contributing to the host’s resistance to vibriosis [86]. For example, a metagenomic analysis utilizing flatfish (*Cynoglossus semilaevis*) as a research model demonstrated that *Phaeobacter* up-regulated its *hdhA* and host *cyp27a1* genes associated with bile acid metabolism while down-regulating its *trxA* gene and the host *akt* gene involved in the proinflammatory cytokine biosynthesis pathways. These findings might suggest the potential role of *Phaeobacter* in mitigating inflammation and enhancing the host’s disease resistance through the microbe–intestine–immunity axis [86].

An important *Phaeobacter* species that can be used as a potential probiotic is *Phaeobacter inhibens* [18]. In a recent study, Panteli, Feidantsis, Demertzioglou, Paralika, Karapanagiotis, Mylonas, Kormas, Mente, Makridis, and Antonopoulou [70] revealed the effect of *P. inhibens* on greater amberjack and reported improved body length and protein synthesis in the metamorphic phase. In this study, hypertrophic growth was indicated by IGF-1/Akt activation and AMPK inhibition. Variations in heat shock proteins (*HSPs*) and reduced MAPKs levels were also evident in the probiotic-treated greater amberjack [70].

The application of the green alga *Ulva ohnoi*, co-cultured with *Phaeobacter* sp. 4UAC3, was effective in reducing the mortality of the *V. anguillarum*-infected turbot larvae of *Scophthalmus maximus*, reared in ‘*Integrated Multi Trophic Aquaculture Recirculation Systems*’ [99]. Although the reduced mortality recorded in the study was not statistically significant, the study indicated the potential of *Phaeobacter*-colonized *U. ohnoi* as an alternative to traditional disease control methods. Roager, Athena-Vasileiadi, Gram, and Sonnenschein [71] demonstrated the in vitro inhibition of the fish pathogens *Vibrio anguillarum* and *V. crassostreae* by both *P. inhibens* DSM17395 and *P. piscinae* S26 in a plate-based assay. Further, *P. piscinae* S26 co-cultured with the microalgae *Tetraselmis suecica* and *Isochrysis galbana* reduced the cell density of pathogenic *V. crassostreae* [71]. Thus, microalgae could be an effective vector to deliver probiotic *Phaeobacter* to fish.

### 4.6. Pseudoalteromonas

Wesseling et al. [100] isolated two different strains of *Pseudoalteromonas* sp. from culture water. They immobilized them on hydrophilized tiles, and their performance against both bacterial (*V. anguillarum*) as well as fungal (*P. lilacinus*) fish pathogens was analysed. Afterwards, functionalized ceramic spawning tiles with probiotic *Pseudoalteromonas* biofilms were designed that could prevent fish egg clutches of the substrate spawners (e.g., clown fishes) from pathogenic infestation, establishing an innovative approach for improved aquaculture. In a succeeding study, *Pseudoalteromonas* sp., either immobilized in alginate beads or grown as a biofilm on ceramic tiles, exhibited anti-*Vibrio* activities against *V. anguillarum*, indicating the potential of both immobilization systems to offer protection against the fish pathogen [101]. Sayes et al. [102] recorded the pathogen inhibitory potential of *Pseudoalteromonas* sp. isolated from the digestive tract of the yellowtail kingfish *Seriola lalandi* and proposed incorporating *Pseudoalteromonas-treated* microalgae fed rotifers and artemia (vectors) in the larval cultures of *S. lalandi to ensure improved larval survival*. A subsequent study by Mejias et al. [103] demonstrated the stimulatory activity of the previously isolated *Pseudoalteromonas* sp. (SLP1) on the growth of the microalgae *Nannochloropsis gaditana*, and the probiotic-supplemented microalgae were incorporated in the mass culture of a rotifer species, *Brachionus plicatilis*. Feeding with *Pseudoalteromonas*-supplemented *N. gaditana* resulted in a significantly greater production of *B. plicatilis* compared to the control (without bacteria supplementation), suggesting the application of *B. plicatilis* as a potential vector to supply probiotic bacteria for the rearing of larval and juvenile *S. lalandi* [103].

The probiotic effects of *Pseudoalteromonas xiamenensis* S1131 were demonstrated using zebrafish as a model organism [104]. The zebrafish larvae (60 h post-fertilization) were pre-exposed to *P. xiamenensis* prior to the pathogenic *Edwardsiella piscicida* challenge, resulting in increased survivability, the suppression of pro-inflammatory markers (*tnfα* and *il6*), and the up-regulation of heat shock protein (*hsp90*) and mucin genes. The antibacterial activity exhibited by *P. xiamenensis* was believed to be correlated with the over-expression of mucin [104]. In a later study, *Pseudoalteromonas ruthenica* S6031 was characterized as an effective probiotic strain that could enhance host defence against *pathogen* infection and thermal stress [73]. Improved tolerance against *E. piscicida* infection, along with increased expressions of immune stress response genes (*muc5.1*, *muc5.2*, *muc5.3*, *alpi2*, *alpi3*, *hsp70*, and *hsp90a*) and the down-regulation of pro-inflammatory genes (*tnfα*, *il1b*, and *il6*) were noticed in *P*. *ruthenica*-immersed zebrafish larvae compared to the control group. Increased resistance against the *E. piscicida* challenge was also recorded in adult zebrafish fed *P. ruthenica*-enriched *Artemia*. Moreover, *P. ruthenica* supplementation revealed an increased abundance of Proteobacteria and Firmicutes, along with a reduced abundance of Bacteroidetes, indicating probiotic-induced modulation in the microbial community within the gut of zebrafish [73]. The cell-free supernatant produced by *Pseudoalteromonas haloplanktis* OS-9 isolated from the rearing surface seawater of a sea bass cage was indicated as a potential *Vibrio* bio-control agent through optimization studies coupled with response surface modelling. *P. haloplanktis* OS-9 was effective against several *Vibrio* spp., exhibiting the most potent inhibition potential against *V. rotiferianus* [105]. In another study, juveniles of sea bass were repeatedly exposed to different strains of *Pseudoalteromonas* spp. (10^6^ CFU/mL; h*Cg*-42 + h*Oe*-125, and RA15), and after that separately challenged with *V. harveyi* and nervous necrosis virus (NNV). Improved survivability of the *D. labrax* juveniles was noticed with *V. harveyi* challenge, while no significant benefit was detected for the NNV challenge [74]. The probiotic effects of live and heat-killed *Pseudoalteromonas piscicida* 2515 were documented in juvenile olive flounder (*Paralichthys olivaceus*) [72]. Both heat-killed (10^5^ CFU/g) and live (10^7^ CFU/g) bacteria exhibited improved non-specific immunity (increased expressions of immune genes) and resistance against *V. anguillarum* infection. Further, supplementation with *P. piscicida* 2515 (live and heat-killed) modulated the microbial community and significantly increased intestinal goblet cell number as well as microvilli length (*p* < 0.05) in olive flounder. Considering the lower dose required to create the probiotic effect, heat treatment was suggested as an effective way to improve probiotic efficiency in fish, both qualitatively (improved immunity and low haemolytic activity) and quantitively (probiotic dose).

### 4.7. Pseudomonas

*Pseudomonads* are common components of the microbiota of fish and freshwater ecosystems and have been widely studied either as a pathogen or for biocontrol purposes in aquaculture [81]. One study evaluates the probiotic potential of GP21 (*Pseudomonas* sp.) and GP12 (*Psychrobacter* sp.), isolated from the gastrointestinal tract of Atlantic cod (*Gadus morhua*). It investigates their antagonistic activity against the fish pathogens *Vibrio anguillarum* and *Aeromonas salmonicida* under various conditions, demonstrating significant antagonistic activity, tolerance to acidic conditions and bile salts, and effective biofilm formation. Both GP21 and GP12 are promising probiotics for enhancing the health and disease resistance of Atlantic cod [75]. *P. fluoresces* biovars I, II, and III were detected as antagonistic against two potential fish pathogens, *P. angulliseptica* and *Streptococcus faecium*, through in vitro agar diffusion assay [80]. Nile tilapia were fed diets containing *P. fluoresces* biovars (10^8^ cells g^−1^) for seven successive days and, after that, challenged with *P. angulliseptica* (3 × 10^7^ cells) or *S. faecium* (3 × 10^8^ cells) by intraperitoneal injection. Following the experimental challenge, fish fed the *P. fluoresces*-incorporated diets exhibited a reduced mortality rate and significant improvement in haematological parameters, total protein, and globulin [80]. Two strains of *P. fluorescens* (LE89 and LE141) isolated from the skin of brown trout and rainbow trout, respectively, were antagonistic to *Saprolegnia parasitica*, causing saprolegniosis in rainbow trout. Increased phagocytic activity of macrophages and serum proteins was recorded with the administration of *P. fluorescens* LE141 (10^6^ bacteria/mL for 6 h, 14 days).

In contrast, the production of siderophores and inhibitory proteins was detected for both *P. fluorescens* LE89 and LE141. Thus, the addition of *P. fluorescens* LE89 and LE141 to the water was suggested for the biocontrol of saprolegniosis. Finally, siderophore production was described as the likely mechanism of action behind the inhibition of *S. parasitica*, although the combination of various mechanisms of action may not be ruled out [81]. Similarly, another study [56] explores bacteria from the gut microbiota of *Seriola lalandi* (yellowtail kingfish). Among the 388 isolates identified, *Shewanella*, *Psychrobacter*, and *Acinetobacter* showed antimicrobial activity against *Vibrio* sp. and stimulated immune-related genes. Despite some *Pseudomonas* isolates showing antibiotic resistance, these findings suggest potential probiotics for disease resistance and immune enhancement in *Seriola lalandi*. In line with these findings, Pacheco, Díaz-Sánchez, Contreras, Villar, Cabezas-Cruz, Gortázar, and de la Fuente [58] investigated the efficacy of high-alpha-Gal-content probiotics in protecting zebrafish against *Mycobacterium marinum*. The probiotics *Aeromonas veronii* and *P. entomophila* were biosafe and significantly reduced mycobacterial infection levels, enhancing immune response and nutrient metabolism while reducing oxidative stress. These probiotics are effective in controlling fish mycobacteriosis and hold potential in broader aquaculture disease management. Li, Jaafar, He, Wu, Kania, and Buchmann [76] assessed the impact of a *Pseudomonas* H6 lipopeptide surfactant on rainbow trout (*Oncorhynchus mykiss*) to control the parasite *Ichthyophthirius multifiliis*. The surfactant demonstrated significant parasiticidal activity and reduced infection rates without adversely affecting the immune response of the host fish, suggesting its use as a biocontrol agent in aquaculture. The potential of *P. entomophila* COFCAU_PEP4 isolated from the intestine of rohu was demonstrated through a series of in vitro evaluations and experimental challenges [79]. The strain revealed antagonistic activities against nine aeromonad indicator strains and *Vibrio parahaemolyticus*, *Escherichia coli*, and *P. aeruginosa*. In addition, a wide pH range (2–9), bile salt tolerance up to 10%, autoaggregation capacity, cell surface hydrophobicity, and non-haemolytic nature were described as probiotic attributes, but validation through in vivo feeding trials or as a water additive merits investigation prior to probiotic application.

Further supporting the potential of *Pseudomonas* species, Qi, Xue, Shi, Wang, and Ling [83] studied the impact of *P. monteilii* JK-1 as an in-feed probiotic on grass carp (*Ctenopharyngodon idella*). The probiotic enhanced growth performance, immune-antioxidant response, and disease resistance. The study of the gut microbiota revealed that feeding with a *P. monteilii* JK-1-supplemented diet had no significant effect on the diversity and bacterial community structure. However, gut microbiota composition was affected. Interestingly, probiotic administration reduced gut microbial disorders caused by *A. hydrophila* infection. These results suggest that *P. monteilii* JK-1 could be an effective probiotic, enhancing growth, immune function, and disease resistance in grass carp. Additionally, Aly, ElBanna, Elatta, Abdel Razek, El-Ramlawy, Mabrok, and Fathi [85] compared the effects of *P. putida* and *Saccharomyces cerevisiae* on the growth, immune response, and disease resistance of Nile tilapia. Both probiotics significantly improved survival rates, growth performance, immune parameters, and resistance against *A. hydrophila* infection, making *P. putida* a promising component in aquaculture feed formulations. Lastly, Lee, Noh, Lee, Hasan, Hur, Lee, Jeong, Lee, Lee, and Kim [77] evaluated host-associated low-temperature probiotics (HALPs) from wild olive flounder (*Paralichthys olivaceus*) intestines. These probiotics significantly improved growth performance, feed utilization, and digestive enzyme activity. The study of gut microbiota revealed that feeding on HALPs significantly affected gut microbiota composition, as noticed by a remarkable increase in beneficial bacteria like *Lactobacillus* and *Lactococcus*. Vinoj, Jayakumar, Chen, Withyachumnarnkul, Shanthi, and Vaseeharan [78] showed that *P. aeruginosa* PsDAHP1 administration to zebrafish for seven days resulted in improved *superoxide dismutase and lysozyme activity and survival towards V. parahaemolyticus*, as well as a decreased colonization of *V. parahaemolyticus on the gills and intestine*. 

Also, in another study, it was reported that *P. fluorescens* supplemented in the diet of rainbow trout showed that probiotic administration affected haematological and biochemical parameters [82]. 

### 4.8. Psychrobacter 

Even though some information is available on *Psychrobacter* as a probiotic in fish, the genus deserves more attention. Current findings indicate that *Psychrobacter* might be capable of producing and secreting antimicrobial compounds [106].

### 4.9. Shewanella

The genus *Shewanella* is widely used as a probiotic in fish, and readers are recommended to have a closer look at the reviews of Cámara-Ruiz, Balebona, Moriñigo, and Esteban [89] and Ringø, Li, Doan, and Ghosh [18]. In recent years, some papers have screened and characterized the potential of Shewanella as a probiotic, and these studies are cited in Table 1.

### 4.10. Vibrio

Some previous studies have used *Vibrio* as a probiotic in fish aquaculture (e.g., [107,108]), even though the majority of *Vibrio* sp. cause diseases. A recent study by Medina, García-Márquez, Moriñigo, and Arijo [90] used *Vibrio proteolyticus* as a probiotic for Senegalese sole (*Solea senegalensis*). This study revealed activated gene expression and improved disease resistance against *V. harveyi* intraperitoneally, but no effect was reported on the *Photobacterium damselae* subsp. *piscicida*.

## 5. Application of Probiotics in Shellfish

Even though the reviews of Ringø [7] and Rohani, Islam, Hossain, Ferdous, Siddik, Nuruzzaman, Padeniya, Brown, and Shahjahan [19] presented some information on probiotic administration in shellfish, in comparison with fish, less information is known about probiotic applications in shellfish. To avoid duplication, studies discussed in the abovementioned reviews are not presented in the text or in Table 2, and readers with an interest in previously published papers are recommended to have a closer look at the studies by Ringø [7] and Rohani, Islam, Hossain, Ferdous, Siddik, Nuruzzaman, Padeniya, Brown, and Shahjahan [19]. Furthermore, readers with an interest in the role of the shrimp gut microbiome in health and disease are recommended to have a closer look at the review by Holt et al. [109].

## 6. Gram-Positives

### 6.1. Clostridium

Sumon et al. [125] demonstrated the effects of *C. butyricum* on the growth digestion and immunity of giant freshwater prawns (*Macrobrachium rosenbergii*) in a 60-day study. They revealed that prawns fed the *C. butyricum*-incorporated diet showed increased specific growth rate, as well as gut protease and amylase activities. Furthermore, prawns fed enriched diets showed increased total, differential, and granular haemocyte count. 

Tadese et al. [126] reported that giant freshwater prawns fed *C. butyricum*-complemented diets showed increased specific growth rate, haemolymph respiratory activity, and nitric oxide synthase levels. Additionally, prawns fed enriched diets showed increased haemolymph TNF-*α*, IL-1, 6, and *IFN-γ* concentrations.

Wangari et al. [127] illustrated the effects of different feeding patterns of dietary *C. butyricum* supplementation on the growth and immunity of giant freshwater prawns and showed that prawns fed the supplemented diets showed increased weight gain and haemolymph *IL-1*, *IL-6*, *TNF-α*, and *IFN-γ* concentrations. However, shrimps fed supplemented diets showed decreased relative mRNA expression levels of *Toll* and *Dorsal*.

The effects of graded levels of *C. butyricum* (10^9^ CFU g^−1^) in the diets of Pacific white shrimp were studied by Duan et al. [128]. The authors reported that the *C. butyricum* groups showed improved growth performance, as well as intestinal amylase and protease activity. In contrast, the lipase activity of the shrimp was only impacted when they were fed with a diet supplemented with 2% *C. butyricum*. Intestinal epithelium height, lysozyme activity, total antioxidant capacity, immunological deficiency gene expression relative level, and Toll gene expression relative level rose in the probiotic groups with the ingestion of *C. butyricum*, and intestinal immune biochemical markers (SOD, CAT, and GPx activate) and genes (HSP70 and ferritin) were expressed at higher levels after exposure to ammonia stress.

Duan et al. [129] reported in a 56-day study on the effects of varying levels of dietary *C. butyricum* (0, 2.5 × 10^9^, 5.0 × 10^9^, and 10^10^ CFU kg^−1^) in Pacific white shrimp. The results revealed that shrimps fed the supplemented diets showed improved gut microbiota composition with increased *Bacillus*, *Clostridium*, *Lachmoclostridium*, *Lachnospiraceae*, and *Lactobacillus* communities in the guts. Moreover, Pacific white shrimp fed supplemented diets showed increased mRNA expressions of digestion (lipase, trypsin, and α-amylase) and immunity-related (lysozyme, crustin, and β-1,3-glucan binding proteins) genes. 

Li et al. [130] fed Pacific white shrimp *C. butyricum* at inclusion levels of 10^7^ to 10^12^ CFU kg^−1^ for 42 days before challenging the shrimp with *V. parahaemolyticus*. The activities of alkaline phosphatase, acid phosphatase, lysozyme, and total nitric oxide synthase in serum were significantly increased, as were growth performance, intestinal villi height, and intestinal wall thickness. However, superoxide dismutase activity was unaffected by the probiotic treatment. The administration of 10^8^–10^12^ CFU kg^−1^ considerably increased the haemolymph’s respiratory burst activity. A significant increase in the survival of treated shrimp exposed to *V. parahaemolyticus* was also seen when 10^11^ and 10^12^ CFU kg^−1^ were included. According to Li, Tian, and Dong [130], *C. butyricum* enhanced intestinal histology in the middle of the gut, immune gene expression, and disease resistance against *V. parahaemolyticus*, as well as growth performance. 

Duan et al. [131] investigated the effects of dietary *C. butyricum* (10^9^ CFU g^−1^) on the growth and intestine digestive enzyme activity of Pacific white shrimp for 56 days. Shrimps were fed diets containing different levels of *C. butyricum* (0, 0.5, 1.0, and 2.0%), and the results showed that feeding with the supplemented diets increased growth and survival rates. Moreover, the intestinal amylase, lipase, and trypsin activities were increased following dietary *C. butyricum* supplementation. 

Luo et al. [132] described the effects of various forms of *C. butyricum* (live cells, sonication-killed cell-free extracts, heat-killed whole-cell and fermented supernatant) on the growth, immunity, and disease confrontation of Pacific white shrimp over 42 days. The results indicated that shrimps fed the *C. butyricum*-supplemented diets showed increased specific growth rates and percentage survival rates. Moreover, Pacific white shrimp fed the supplemented diets showed improved lysozyme and peroxidase activities. Also, the mRNA expressions of the *LZM*, *proPO*, *LGBP*, *HSP70*, *Imd*, *Toll*, *Relish*, *TOR*, *4E-BP*, *eIF4E1α*, and *eIF4E2* genes increased in the hepatopancreas of the shrimps fed probiotic-supplemented diets.

In a recent study, Liang, Tran, Deng, Li, Lei, Bakky, Zhang, Li, Chen, and Zhang [110] investigated the effect of *C. butyricum* supplementation on mud crabs (*Scylla paramamosain*) and revealed improved resistance against *V. parahaemolyticus*. In addition, the supplementation affected the abundance and diversity of the microbiota sampled from the gut contents of the posterior intestine.

In a 56-day experiment, Duan et al. [133] reported that the administration of *C. butyricum* to kuruma shrimps (*Marsupenaeus japonicus*) modulated their intestinal digestive and metabolic capacities by increasing intestinal pepsin, 5-hydroxytryptamine, amylase and lipase activities, and intestinal propionic, as well as butyric acid and crude protein at the highest inclusion level (200 mg g^−1^).

### 6.2. Microbacterium

Zheng, Yu, Liu, Su, Xu, Yu, and Zhang [111] tested the probiotic potential of *Microbacterium aquimaris* isolated from Pacific white shrimp and concluded that the bacterium is a potential probiotic candidate in shrimp hatcheries. 

### 6.3. Paenibacillus

Amoah, Huang, Dong, Tan, Zhang, Chi, Yang, Liu, and Yang [112] evaluated the effect of *P. polymyxa* ATCC 842 administration on Pacific white shrimp; they found that it increased resistance against *V. parahaemolyticus*, and the abundance and diversity of the microbiota sampled from the gut contents of the posterior intestine as beneficial bacteria (*Ruegeria* and *Pseudoalteromonas*) were significantly enhanced in the probiotic-treated group vs. the control, while opportunistic bacterial pathogens (*Vibrio*, *Photobacterium*, *Tenacibaculum*, and *Shewanella*) significantly decreased. 

## 7. Gram-Negatives

### 7.1. Aeromonas 

Little information is available on *Aeromonas* as a probiotic in shellfish aquaculture. However, in an early study, Gibson, Woodworth, and George [97] tested the probiotic ability of the *Aeromonas media* strain A199, revealing antagonistic activity against several pathogens at 10^4^ CFU mL^−1^ in Pacific oyster (*Crassostrea gigas*) challenged with *Vibrio tubiashii*, with a significant effect on survival after five days.

### 7.2. Enterobacter

Collectively, these studies highlight the promising potential of various probiotic strains as sustainable alternatives to antibiotics in aquaculture. In a previous study, LaPatra, Fehringer, and Cain [64] demonstrated that the *Enterobacter* sp. strain C6-6 significantly enhances immune response in rainbow trout, providing substantial protection against *Flavobacterium psychrophilum* and effectively reducing mortality rates. Similarly, Zakaria, Yaminudin, Yasin, Ikhsan, and Karim [65] reported that *Enterobacter* sp. G87 significantly inhibits *Vibrio anguillarum*, enhancing survival rates and immune responses in fish while reducing histopathological damage. Additionally, Amin, Bolch, Adams, and Burke [114] showed that probiotic supplementation, particularly with *Bacillus amyloliquefaciens* MA228 and *Enterobacter ludwigii* MA208, significantly improves growth performance and feed efficiency in juvenile abalone.

Furthermore, Suryaningsih, Maulana, Istiqomah, and Isnansetyo [67] reported that *Bacillus* sp. (PCP1) and *Enterobacter* sp. (JC10) exhibit strong adhesion to intestinal cells in red tilapia. However, their short-term application did not significantly affect growth or survival, suggesting the need for longer-term studies. Together, these findings underscore the effectiveness of probiotics in enhancing fish health and growth, supporting more sustainable and antibiotic-free aquaculture practices.

### 7.3. Paenibacillus

The collective body of research on probiotics in aquaculture underscores their profound benefits in promoting fish health, growth performance, immune response, and disease resistance, thereby providing a sustainable alternative to antibiotics. For instance, Gupta, Gupta, and Dhawan [51] revealed that *P. polymyxa* as a water additive significantly enhanced the survival rates and innate immune responses of common carp, particularly at concentrations of 10^3^ and 10^4^ CFU/mL. This study underscores the potential of *P. polymyxa* in improving disease resistance without compromising water quality, a critical factor for sustainable aquaculture practices. Building on this, Chen, Liu, and Hu [49] demonstrated that dietary supplementation with *Paenibacillus ehimensis* NPUST1 significantly boosted the growth performance, feed efficiency, and immune responses of Nile tilapia. These enhanced survival rates and immune parameters, including increased phagocytic and respiratory burst activities, further highlight the probiotic’s role in promoting overall fish health and resilience against pathogens like *A. hydrophila* and *S. iniae*. In another study, Midhun, Arun, Neethu, Vysakh, Radhakrishnan, and Jyothis [52] reported that *P. polymyxa* HGA4C significantly improved growth parameters, digestive enzyme production, and antioxidant enzyme activities in Nile tilapia. The upregulation of growth-related and immune-related genes suggests that *P. polymyxa* HGA4C can enhance both immune status and feed utilization efficiency, which is crucial for the sustainable development of aquaculture. Amoah, Huang, Dong, Tan, Zhang, Chi, Yang, Liu, and Yang [112] evaluated the benefits of *P. polymyxa* ATCC 842 in Pacific white shrimp, finding significant improvements in growth performance, immune response, and disease resistance against *V. parahaemolyticus*. This research supports the use of *P. polymyxa* as a promising alternative to antibiotics in shrimp aquaculture, aligning with global efforts to reduce antibiotic use in food production.

Further supporting these findings, Lin, Chen, Wen, and Hu [50] showed that *Pediococcus acidilactici* NPUST1 enhanced the innate immunity and disease resistance of zebrafish, along with positive effects on glucose metabolism. This highlights the broad applicability of probiotics in various aquaculture species, potentially improving overall fish health and sustainability. Yang, Jin, Li, Jiang, Cui, Huang, Rang, Li, and Xia [53] introduced a novel strain of *P. polymyxa* S3, which improved growth, immune-related enzyme activities, and survival rates in grass carp (*Ctenopharyngodon idellus*) against *A. hydrophila*. The strain’s antagonistic activity against major fish pathogens further underscores its potential as a robust probiotic in aquaculture. In juvenile northern whiting fish (*Sillago sihama* Forsskál), Amoah, Dong, Tan, Zhang, Chi, Yang, Liu, Yang, and Zhang [113] reported that *Bacillus coagulans*, *Bacillus licheniformis*, and *P. polymyxa* significantly improved growth performance, feed utilization, immune response, and resistance to *Vibrio harveyi* infection. These probiotics showed promise as effective alternatives to antibiotics, promoting healthier and more resilient fish populations. Liao, Huang, Lin, Chen, Lee, Lin, Chuang, and Nan [54] studied the impact of commercial probiotics on Asian seabass (*Lates calcarifer*), demonstrating improvements in growth parameters, non-specific immune responses, and disease resistance against *Vibrio alginolyticus*. These findings support the integration of probiotics into aquaculture practices to enhance fish health and sustainability. Finally, Jose, Arun, Neethu, Radhakrishnan, and Jyothis [55] explored the effects of a bacterial consortium of *P. polymyxa* HGA4C and *Bacillus licheniformis* HGA8B on Nile tilapia. The consortium enhanced growth performance, feed utilization, enzyme production, and immune responses, while also improving resistance to *A. hydrophila*. This study underscores the potential of probiotic combinations as eco-friendly alternatives to antibiotics, fostering better growth and health in aquaculture species.

### 7.4. Phaeobacter

In his review of probiotics in shellfish aquaculture, Ringø [7] reported and discussed the use of *Phaeobacter*. To avoid overlaps, these studies are not discussed in the present review. Zhao et al. [134] revealed that *Phaeobacter inhibens* downregulated virulence factor transcription in the shellfish pathogen *Vibrio coralliilyticus* via N-acyl homoserine lactone production. In a subsequent study, Zhao et al. [135] investigated the benefits of *Phaeobacter daeponensis* administration to abalone (*Haliotis diversicolor*) for 180 days. They revealed that the bacterium significantly improved shell length, wet weight, immunological function, and disease resistance to *V. harveyi*. The study of gut microbiota modulation revealed that treatment with the probiotic significantly increased the richness of beneficial endogenous bacteria such as bacilli and actinobacterial species. Also, feeding with *P. daeponensis* helped abalone to re-establish and balance the gut microbiota post-challenge with *V. harveyi*. Among the genera under *Phaeobacter*, *P. inhibens* has been recommended as a probiotic bacterium in marine aquaculture systems. In an early study, it was revealed that *Phaeobacter inhibens* stimulate growth and enhance disease resistance to the pathogenic bacteria *V. vulnificus* in European flat oysters (*Ostrea edulis*) [115].

In another study, the addition of *P. inhibens* S4 to culture tanks did not significantly affect the survival and growth of eastern oyster (*Crassostrea virginica*) larvae, although increased survivability in the probiotic-treated (10^4^ CFU/mL) larvae was noticed following experimental infection with the bacterial pathogen *Vibrio coralliilyticus* RE22 [116]. A later study documented the effects of probiotic *P. inhibens* S4 on the microbial communities of *C. virginica* larvae [117]. *P. inhibens* treatment was associated with significant changes in the relative abundances of 18 amplicon sequence variants (ASVs), including 2 abundant ASVs in *Alteromonas* and *Pseudomonas*, suggesting ASV-specific effects of the probiotic on the larval bacterial community.

### 7.5. Pseudoalteromonas

*Pseudoalteromonas* spp. strains encompass widely distributed heterotrophic, flagellated, non-spore-forming, rod-shaped, and Gram-negative marine probiotic bacteria, with up to 37 to 48 specified species [136]. Diverse strains of *Pseudoalteromonas* spp. are prevalent in nature and can diminish competing microflora. The application of *Pseudoalteromonas* sp. D41 has been recommended as a potential probiotic in mollusc larviculture [137]. The prior administration of *Pseudoalteromonas* sp. D41 in the rearing water of scallop (*Pecten maximus*) larvae at 10^3^ CFU ml^−1^ resulted in 35% better survivability than the pathogen control against *V. splendidus*, while it was ineffective against *V. coralliilyticus*. In Pacific oyster (*Crassostrea gigas*), *Pseudoalteromonas* sp. D41 was effective against the challenge by *V. coralliilyticus* (50% improved survival) but not against *V. pectenicida* [137]. The supplementation of *Pseudoalteromonas* sp. BC228 in the diets of juvenile sea cucumbers (*Apostichopus japonicus*) improved their digestive enzymes (trypsin and lipase), stimulated phagocytic activity in the coelomocytes, enhanced lysozyme and phenoloxidase activities in the coelomic fluid, and offered resistance against *V. splendidus* infection [122]. Ma, Liu, Li, Tao, Yu, and Liu [122] appraised the effects of the probiotic *Pseudoalteromonas* sp. F15 on growth, survival, and digestion, as well as on immune-related enzyme activities in the larvae and juveniles of the Yesso scallop, *Patinopecten yessoensis*. Scallop larvae and juveniles were fed live microalgae (*Dicrateria inornata*, *Nitzschia Closterium*, and *Platymonas helgolandica*), and a probiotic suspension (10^4^ and 10^6^ cells mL^−1^) was added to the water. Significant increases in the activities of digestive (pepsin, amylase, and cellulase) as well as immune enzymes (lysozyme, superoxide dismutase, and catalase) were noticed in the larvae and juveniles upon *Pseudoalteromonas* sp. F15 supplementation compared to the groups fed only microalgae, indicating the efficacy of the probiotic strain in scallop larviculture [122]. Around 54% of the *Pseudoalteromonas* strains isolated from the haemolymph of marine molluscs (oysters and mussels) exhibited antimicrobial activity against *Vibrio harveyi* ORM4 [138]. Afterward, an immersion treatment in a seawater suspension of *Pseudoalteromonas* h*Cg*-6 (1 × 10^6^ CFU mL^−1^) exhibited protective efficacy on abalone (*Haliotis tuberculate*) challenged with the *Vibrio harveyi* ORM4 strain, suggesting the use of the *Pseudoalteromonas* h*Cg*-6 strain to prevent *Vibrio* infection in abalone culture [138]. *Pseudoalteromonas piscicida* 2202 isolated from the haemal fluid of the bivalve mollusc *Modiolus kurilensis* demonstrated selective behaviour against diverse pathogenic bacteria and larvae of various invertebrates. It was antagonistic against *Staphylococcus aureus*, *Candida albicans*, and *Bacillus subtilis*, but not against *E. coli* and *P. aeruginosa*. Further, *P. piscicida* 2202 displayed selective toxicity and impaired the early development of *Mytilus edulis*, but not of *Strongylocentrotus nudus* [120]. Such species-specific patterns of interaction must be considered when using a putative probiotic bacterium to culture any aquatic species. 

Yuhana and Zairin Jr [139] recorded the protease, amylase, lipase, and mannanase-producing ability of the probiotic *Pseudoalteromonas piscicida* 1Ub. In their study, the enrichment of *Artemia* sp. with *P. piscicida* 1Ub (10^6^ CFU mL^−1^) alone or in combination with the prebiotic (as synbiotic) mannan-oligosaccharide (12 mg L^−1^) improved the nutritional value as well as the bacterial population of the *Artemia* sp. [139]. A strain of *Pseudoalteromonas* sp. NC201, isolated from the coastal environment of New Caledonia, offered protection to the Pacific blue shrimp (*Litopenaeus stylirostris*) against biotic and abiotic stresses. Subadults of Pacific blue shrimp were exposed to *Pseudoalteromonas* sp. NC201 (10^5^ CFU L^−1^) on alternate days throughout the rearing period, and then were challenged with *Vibrio nigripulchritudo* (10^5^ CFU mL^−1^). The probiotic-treated shrimp showed reduced cumulative mortality, lowered pathogen prevalence, and lesser lysozyme transcript numbers, along with an increased survival rate against hyposaline stress [140]. In another study, *Pseudoalteromonas* spp. strains (CDM8 and CDA22) isolated from the hindgut of healthy Pacific white shrimp displayed antagonism against *V. parahaemolyticus*, causing acute hepatopancreatic necrosis disease in shrimps [31]. Diets supplemented with *Pseudoalteromonas* spp. (10^7^ CFU kg^−1^) for 21 days reduced cumulative mortality and significantly decreased the presumptive *Vibrio* counts in the hindgut of Pacific white shrimp experimentally challenged with *V. parahaemolyticus*. In addition, decreased copy numbers of the toxin gene pirABvp in *V. parahaemolyticus* were also recorded [31]. The administration of *Pseudoalteromonas piscicida* 1UB for 40 days, with or without FOS, improved growth, immunity, and protection against white spot syndrome virus and *V. harveyi* coinfection in Pacific white shrimp [121]. In this study, the increase in total haemocyte count, phenol-oxidase, respiratory burst activity, and immune-related gene expression after coinfection was associated with a significant reduction in shrimp mortality. Similar results were achieved with Pacific white shrimp fed diets supplemented with synbiotic microcapsules consisting of *P*. *piscicida* 1UB and/or *Bacillus* NP5 as probiotics (10^8^ CFU g^−1^), along with mannan-oligosaccharides (MOS) as a prebiotic. Synbiotic supplementation significantly improved growth, microbial diversity, non-specific immune parameters (respiratory burst, phenoloxidase, and phagocytic activity), and resistance to *V. parahaemolyticus* infection, and the best result was recorded with the synbiotics containing *P. piscicida* 1UB [141].

In contrast, the application of *Pseudoalteromonas flavipulchra* (10^8^ CFU mL^−1^) could not support the population growth of the rotifer (*B. plicatilis*), although it effectively suppressed *Vibrio* in culture [118]. Similarly, the poor growth of *Artemia franciscana* nauplii associated with the reduced retention of the probiotic bacterium was recorded for *P. flavipulchra* (10^8^ CFU mL^−1^) added to the experimental culture, even though the suppression of *Vibrio* was evidenced [119].

In addition to finfish and shellfish culture, the efficacy of *Pseudoalteromonas* sp. has also been evaluated in marine algae culture. The co-inoculation of a strain of *Pseudoalteromonas* sp. PB2-1 along with *Phaeobacter* sp. BS52 could prevent pathogen-induced bleaching disease in two red macroalgae: *Delisea pulchra* and *Agarophyton vermiculophyllum* [142]. 

### 7.6. Pseudomonas

In a previous study, equal combinations (10^5^ CFU mL^−1^) of both *P. synxantha* and *P. aeruginosa* in the diet for 84 days improved the specific growth rate, survival, and immune parameters of juvenile western king prawns, *Penaeus latisulcatus* [143].

In a more recent study, *P. putida* and three other commercial probiotic species (*Lactobacillus plantarum*, *Lactobacillus fermentum*, and *Bacillus subtilis*) were applied to commercial shrimp culture ponds, and the presence of these probiotic strains in the rearing water and intestine of the shrimp were evaluated on day 47 through high-throughput sequencing. None of the commercial probiotic species, including *P. putida*, were detected in the rearing water or shrimp digestive tracts, suggesting the low viability and adaptability of the applied probiotic strains in the rearing pond as well as in the shrimp intestines [124].

### 7.7. Vibrio

As several species within the genus *Vibrio* cause fish diseases, little information is available regarding *Vibrio* as a probiotic in shellfish aquaculture. However, the information available is presented in [7]. 

## 8. Conclusions and Future Perspectives 

Considering the literature reviewed, it is concluded that probiotic species beyond Bacillus and Lactobacillus, particularly those isolated from host organisms or culture environments, demonstrate significant benefits against pathogenic and opportunistic bacteria. They can be considered a promising means of disease prevention and treatment. Also, given the fact that these microbes are indigenous to the host, the administration of host-associated probiotics minimizes biosecurity risks. Although there is available information on the effects of these probiotics, there are very limited commercial products in this context. Also, there is a gap in the existing knowledge about the development of multi-strain mixtures and consortia of probiotics. The application of a consortium of probiotics has been shown to be more effective than single-strain probiotics, and thus must be considered in cases of probiotics other than LAB and non-LAB. Given the fact that providing a substrate for the growth of probiotic bacteria can sustain their colonization in the gastrointestinal tract, the study of optimum prebiotics as a substrate for each isolated non-LAB to form novel and efficient synbiotics should be considered in future.

## Figures and Tables

**Table 1:** Effects of promising probiotics on growth performance, immune response, and disease resistance in fish.

Bacterial Species	Doses	Duration (Days)	Finfish Species	Parameters Investigated	References
**Gram-positives**					
*B. animalis* subsp. *Lactis*	10^10^ cells g^−1^	15 or 30	Japanese seabass (*Lateolabrax japonicus*)	↑ Growth, serum antioxidant capacity and innate immunity; modulated hindgut microbiota; ↓ levels of oxidants	[23]
*B. bifidum* and *Lactobacillus acidophilus*	0, 1, 2, and 3 g kg^−1^	56	Hybrid catfish (*Heteroclarias*)	↑ Growth performance and survival	[24]
*B. bifidum*	10^7^ cells/100 g diet	98	Nile tilapia (*Oreochromis* *niloticus*)	↑ Growth performance and resistance against *Aeromonas hydrophila*	[25]
*B. animalis* and *B. lactis*	10^7^, 2 × 10^7^, and 3 × 10^7^	56 days	Rainbow trout (*Oncorhynchus mykiss*)	By feeding the lowest supplementation, highest growth performance and gut lactobacilli were observed	[26]
*B. bifidum*, *B. breve*, *B. lactis* and different species of lactic acid bacteria	3.2 × 10^9^ CFU g^−1^	N/A	Common carp (*Cyprinus carpio*)	↑ Growth performance and haematological profile	[27]
*B. bifidum*, *Enterococcus faecium*, different species of lactobacilli and *Pediococcus acidilactici*	10^9^ CFU g^−1^	90	Nile tilapia	↑ Growth affects muscle growth and gene expression, and increases the number of intestinal villi	[28]
*B. bifidum*, *Lactobacillus* sp. and *B. subtilis*	10^6^, 2 × 10^6^, and 3 × 10^6^ kg^−1^	56	Siberian sturgeon (*Acipenser baerii*)	↑ Growth performance, lysozymes, and IgM	[29]
*B. bifidum* and *Lactobacillus acidophilus*	0.5 and 1.0 g kg^−1^	56	Rainbow trout	↑ Growth and feed conversion, serum complement, lysozyme, and bactericidal activities, and resistance against *Yersinia ruckeri*	[30]
*B. lactis* and *Lactobacillus*	5 × 10^6^ CFU g^−1^	56	Asian seabass (*Lates calcarifer*)	↑ Growth performance, microvilli length, total amino acids in muscle, and resistance against *Streptococcus iniae*; modulated the gut microbiota by decreasing pathogens	[31]
*B. longum*, *B. thermophilum*, *Bacillus subtilis*, and *Lactobacillus acidophilus*	0, 1, 2, 3, and 4 g bacteria mixture kg^−1^	90	Nile tilapia	↑ Growth performance and fish health	[32]
*Bifidobacterium*, *Lactobacillus*, *Saccharomyces cerevisiae*, *Spirulina*, and phytase	0.5, 1, and 2 g kg^−1^	Hatchlings (day 8–38), fry (day 38–68), and advanced fry (day 68–98)	Rohu (*Labeo rohita*)	↑ Survival and specific growth rate	[33]
*Bifidobacterium* sp., *L. acidophilus*, and *E. faecium*	3.5 × 10^9^ CFU g^−1^ *Bifidobacterium* sp., 3.5 × 10^9^ CFU g^−1^, *L. acidophilus*, and 3.5 × 10^9^ CFU g^−1^ *E. faecium*	63	Nile tilapia	↑ Resistance against *A. hydrophila* without growth reduction	[34]
*Brevibacillus brevis*	*Artemia* as vector	N/A	European seabass (*Dicentrarchus labrax*) larvae	↑ Growth	[35]
*B. brevis*	10^7^ CFU mL^−1^	N/A	Rainbow trout	Probiotic administration affected haematological and biochemical parameters	[36]
*Brevibacillus laterosporus*	5 × 10^3^, 5 × 10^4^, and 5 × 10^5^ CFU mL^−1^	56	Crucian carp (*Carassius auratus*)	↑ Water quality, growth performance, antioxidant capacity, and digestive enzyme activities	[37]
*Clostridium autoethanogenum* protein (CAP)	Dose N/A	56	Largemouth bass (*Micropterus salmoides*)	↑ Growth and intestinal health and modulated the gut microbiota	[38]
Combination of *T. molitor-*, *Chlorella* meal, CAP, and cottonseed protein concentrate at a ratio of 1:1:6:4 (Blend B)	Dose N/A	56	Gibel carp (*Carassius gibelio*)	↑ Growth and intestinal health	[39]
*Clostridium butyricum*	10^6^ CFU g^−1^	10	Crucian carp	Administration modulated the gut microbiota and improved the microbial metabolism	[40]
*C. butyricum*	10^8^ CFU g^−1^	56	Chinese perch (*Siniperca chuasti*)	↓ Growth performance, length of intestinal villi affecting nutrient absorption efficiency, and gut microbial diversity	[41]
*C. butyricum*	10^6^, 10^7^ (CB2), and 10^8^ CFU g^−1^	56	Turbot (*Scophthalmus maximus*)	Administration of CB2 ↑ growth, thickness, width, and height of intestinal epithelium and up-regulation of tight junction proteins; modulated the gut microbiota	[42]
*C. butyricum*	Doses are presented in the review	Durations are presented in the review	Different fish species	The review described health effects and disease resistance	[43]
*C. butyricum*	0.1 × 10^7^, 2 × 10^7^, 3 × 10^7^, and 4 × 10^7^ CFU g^−1^	56	Hybrid grouper (*Epinephelus lanceolatus*♂ × *E. fuscoguttatus*♀)	↑ Growth performance, intestinal enzyme activities, and intestinal morphology; affected expression levels in the intestine of antioxidant-related genes, immune-related genes, tight junction protein genes, and intestinal microbiota	[44]
*Microbacterium*, *Ruegeria*, *Pseudomonas*, and *Vibrio*	5 × 10^6^ CFU ml^−1^	10	Atlantic cod (*Gadus morhua*) larvae	Only *Microbacterium* seems to colonise the larval intestine even though all candidates originated from cod larvae intestine	[45]
*Microbacterium* sp. and *Rummeliibacillus* sp.	10^8^ CFU g^−1^ (50:50 ratio)	56	Flounder (*Paralichthys olivaceus*)	↑ Growth, feed utilisation, the immune parameter myeloperoxidase, and the abundance of beneficial gut bacteria → serum biochemical parameters	[46]
*Micrococcus aloeverae*	10^8^ cells g^−1^	42	Japanese seabass	→ Weight gain and specific growth rate	[47]
*Micrococcus yunnanensis*	10^8^ cells g^−1^	42	Japanese seabass	↑ Weight gain and specific growth rate	[47]
*Micrococcus luteus*	In vitro test	_	Isolated from tiger grouper (*Epinephelus fuscoguttatus*)	Revealed antagonistic activity against four pathogens	[48]
*Paenibacillus ehimensis* NPUST1	10^6^ and 10^7^ CFU g^−1^	60	Nile tilapia	↑ Growth performance and innate immunity; ↑ disease resistance against *A. hydrophila* and *S. iniae*	[49]
*Paenibacillus ehimensis*	10^6^ and 10^7^ CFU g^−1^	56	Zebrafish (*Danio rerio*)	↑ Hepatic mRNA expression of carbohydrate metabolism-related genes and innate immune-related genes, and resistance against *A. hydrophila* and *S. iniae*	[50]
*Paenibacillus polymyxa*	10^3^ (PP1), 10^4^ (PP2), and 10^5^ CFU mL^−1^ (PP3)	60	Common carp	↑ Growth performance, innate immunity, and disease resistance against *A. hydrophila*	[51]
*P. polymyxa* HGA4C	10^6^ and 10^8^ CFU g^−1^	60	Nile tilapia	↑ Growth performance and immune response; upregulated the expression of growth and immune-related genes; intestinal MUC 2 up-regulation showed mucosal remodelling in the fish	[52]
*P. polymyxa*	10^6^ cells mL^−1^	30	Grass carp (*Ctenopharyngodon idellus*)	In vitro test showed that the bacterium displayed antagonistic activity against 11 pathogens; colonised the abdomen; ↑ growth and enzyme activities; upregulated the expression of antioxidant-related genes and immune-related genes; resistance against *A. hydrophila*	[53]
*Paenibacillus* sp., *B. subtilis*, *Bacillus amyloliquefaciens*, and *Lactobacillus rhamnosus*	10^7^ CFU g^−1^ of each probiotic	56	Asian seabass	↑ Growth performance and resistance against *Vibrio alginolyticus*	[54]
*P. polymyxa* HGA4C and *Bacillus licheniformis* HGA8B	10^6^ (PB1) and 10^8^ CFU g^−1^ (PB2)		Nile tilapia	↑ Growth performance, immune response, and upregulated expression of growth- and immune-related genes; ↑ intestinal MUC 2 up-regulation showed mucosal remodelling in the fish and disease resistance against *A. hydrophila*	[55]
*Gram-negatives*					
*Acinetobacter* sp. P27 and P33	In vitro test of a new potential probiotic bacteria	_	Isolated from intestinal content of wild great amberjack (*Seriola lalandi*)	↑ Antimicrobial activity against *Vibrio* 25LT1	[56]
*Aeromonas veronii* V03	3.2 × 10^7^ and 3.5 × 10^9^ CFU g^−1^	28	Common carp	↑ Growth, innate immunity, and resistance against *A. hydrophila*	[57]
*A. veronii* (with high α-Gal content)	10^6^, 10^7^, and 10^8^ CFU fish^−1^ (injected intra-peritoneally	7	Zebrafish	Modified the gut microbiota and innate immune responses; beneficial effect on nutrient metabolism and reduced oxidative stress; Effective to control *Mycobacterium marinum*	[58]
*Alcaligenes faecalis* subsp. *faecalis*	In vitro characterization of a new potential probiotic bacteria	_	Isolated from intestinal contents of meagre (*Argyrosomus regius*)	Potentially probiotic due to production of antibacterial substances, resistance to pH gradients, adhesion, growth in mucus, resistance to bile, hydrophobicity, and competition for nutrients	[59]
*Alcaligenes faecalis*	Doses are presented in the review	Durations are presented in the review	Different fish species	The authors stated “*due to its antimicrobial properties it can act as probiotics and can often be used as biocontrol agent*”	[60]
*Alcaligenes faecalis* Y311	10^4^ cells mL^− 1^ added every 7 days	90	Nile tilapia	↑ Intestinal alkaline phosphatase activities; → abundance of the main gut bacteria; ↓ abundance of potential pathogens	[61]
*Alcaligenes* sp. AFG22	10^8^ CFU g^−1^	90	Malaysian Mahseer (*Tor tanbroides*)	↑ Villus length, villus with, villus area, and number of lipolytic, proteolytic, and cellulolytic bacteria	[62]
*Alcaligenes* sp. AFG22	10^8^ CFU g^−1^	90	Malaysian Mahseer	↑ Growth performance and upregulated growth-related gene expression and hypertrophic muscle progression	[63]
*Enterobacter* sp.	10^7^ CFU mL^−1^	56	Rainbow trout	↑ Disease resistance and innate and adaptive immunity	[64]
*Enterobacter* sp. G87	10^4^, 10^5^, and 10^6^ CFU mL^−1^		Asian seabass	↑ Growth performance and disease resistance	[65]
*Enterobacter asburiae* E7	10^7^ CFU g^−1^	28	Common carp	Revealed antibacterial activities against 12 pathogens; upregulation of immune-related genes; ↑ resistance against *Aeromonas veronii;* → growth	[66]
*Enterobacter* sp. (JC10) and *Bacillus* sp. (PCP1)	5 × 10^5^ CFU g^−1^	30	Nile tilapia	→ Growth performance	[67]
*Phaeobacter*	Rotifers and Artemia used as vectors, 5 × 10^7^ CFU mL^−1^	60	European seabass larvae	↑ Specific growth rate and bacterial diversity, but did not appear after probiotic administration stopped after 18 days	[68]
*Phaeobacter*	Doses are presented in the review	Durations are presented in the review	Different fish species	The review described health effects and disease resistance	[18]
*Phaeobacter* sp. and *Phaeobacter gallaeciensis*	Characterisation of host-associated microbiota	_	Phaeobacter was isolated from greater amberjack (*Seriola dumerili*) and *Artemia*	Inhibited in vivo growth of *A. veronii*, *Vibrio harveyi*, *Vibrio anguillarum*, and *V. alginolyticus*	[69]
*Phaeobacter inhibens*	N/A, water additive	N/A	Greater amberjack	Entered the metamorphic phase with greater body length; protein synthesis was triggered to facilitate hypertrophic growth	[70]
*Phaeobacter piscinae* strain S26	Testing antagonistic activity	_	Isolated from Greek seabass larval unit	S26 produces the antibacterial compound tropodithietic acid; as S26 was more effective than *P. inhibens* in inhibition of pathogens, the author suggested S26 as a promising new probiotic candidate	[71]
*Pseudoalteromonas piscicida* 2515	10^6^, 10^7^, 10^8^, and 10^9^ CFU g^−1^	35	Olive flounder (*Paralichthys olivaceus*)	↑ Immune system and disease resistance against *V. anguillarum;* modulated intestinal microbiota	[72]
*Pseudoalteromonas ruthenica*	3.4 × 10^8^ CFU mL^−1^	N/A	Zebrafish larvae	↑ Resistance against *Edwardsiella piscicida;* low pro-inflammatory and high responsive protein expression levels; improved goblet cell density and average villi height; modulated the gut microbiota	[73]
*Pseudoalteromonas*, mixed strains (hCg-42+hOe-125)	10^6^ CFU mL^−1^	56-86	Seabass	↑ Resistance against *V. harveyi*	[74]
*Pseudomonas* sp. GP21	10^8^ cells mL^−1^	3 and 24 h	Head kidney leukocytes of Atlantic cod	↑ Defence genes BPI/LBP and g-type lysozyme, cytotoxic cell receptor protein-1 (NCCRP-1), and GSH-Px	[75]
*Pseudomonas* sp. P18	In vitro evaluation of potential probiotics	_	Isolated from great amberjack	↑ Antimicrobial activity against *Vibrio* 25LT1, *Vibrio* 25LS1, and *Vibrio* 25LH1	[56]
*Pseudomonas H6* surfactant	10 mg/L	10	Rainbow trout	↑ Disease resistance against *A. hydrophila*	[76]
*Pseudomonas* species	10^8^ CFU/g	60	Olive flounder	↑ Growth performance, digestive enzyme activity, and gut microbiota composition → Growth, immunity, and apoptosis-related gene expression	[77]
*P. aeruginosa* PsDAHP1	Intestine of healthy Indian shrimp (*Fenneropenaeus indicus*)	10	Zebrafish	↓ Colonization of *Vibrio parahaemolyticus on gills and intestine;* ↑ *superoxide dismutase* and *lysozyme* activity and survival against *V. parahaemolyticus*	[78]
*P. entomophilia* (with high α-Gal content)	10^6^, 10^7^, and 10^8^ CFU fish^−1^ (injected intra-peritoneally)	7	Zebrafish	Modified the gut microbiota and innate immune responses; beneficial effect on nutrient metabolism and reduced oxidative stress; effective to control *Mycobacterium marinum*	[58]
*P. entomophilia*	Characterization of potential probiotics	_	Isolated from rohu intestine	The strain revealed antagonistic effect towards 12 pathogenic bacteria; tolerated high pH and bile concentrations; in vitro mucosal adherence, auto-aggregation capacity, and production of extracellular enzymes	[79]
*P. fluorescents*	10^8^ CFU g^−1^	14	Nile tilapia (*Oreochromis niloticus*)	↑ Haematological parameters, total protein, and globulin ↓ mortality against two fish pathogens *P. angulliseptica* and *Streptococcus faecium*	[80]
*P. fluorescens* strains LE89 and LE141	10^6^ CFU mL^−1^	14	Rainbow trout	↑ Innate immune response, the production of siderophores, phagocytic activity; ↓ *Saprolegnia parasitica* infection	[81]
*P. fluorescens*	10^7^ CFU mL^−1^	N/A	Rainbow trout	Probiotic administration affected haematological and biochemical parameters	[82]
*P. monteilii*	10^8^ CFU g^−1^	56	Grass carp	↑ Growth performance, expression of immune-related genes, antioxidant enzymes and disease resistance; ↓ *Aeromonas* load in liver and gut; modulated the gut microbiota	[83]
*P. mosselii*	Characterization of potential probiotics	_	Isolated from rohu intestine	Revealed antagonistic effect towards 14 pathogenic bacteria; tolerated high pH and bile concentrations; in vitro mucosal adherence, auto-aggregation capacity, and production of extracellular enzymes; sensitive to several antibiotics	[84]
*P. putida*	10^7^ CFU g^−1^	60	Nile tilapia	↑ Growth performance, immune response and disease resistance against *A. hydrophila*	[85]
*Shewanella coralllii*	Screening and characterization of potential probiotics	_	Isolated from hybrid grouper intestine	Based on simulated gastro-intestinal fluid tolerance, adhesion, digestive enzyme production, antibacterial activity and no signs of disease symptoms or death in grouper, the authors suggested probiotic potential	[86]
*c putrefaciens*	*S. putrefaciens* Pdp11 is a well-known strain used as a probiotic in aquaculture	_	Isolated from diseased eels	The paper describes that two of five pathogenic strains of *S. putrefaciens* contain plasmids, but no plasmids were revealed in the probiotic Pdp11 strain	[87]
*S. putrefaciens* Pdp11	Postbiotic use of bacterial metabolites including extracellular products (ECPs), improving host physiology	_	N/A but was selected due to in vitro and in vivo ability	The investigation evaluates how ECPs are affected by culture media, cultivation temperature, growth phase, and salinity	[88]
*Shewanella*	Doses are presented in the reviews	Durations are presented in the reviews	Different fish species	The reviews describe health effects and disease resistance	[89]
*Vibrio proteolyticus*	Injected intra-peritoneally using 0.1 mL of 10^9^ CFU mL^−1^, bath, or diet	30	Senegalese sole (*Solea senegalensis*)	Activated gene expression; ↑ disease resistance against intraperitoneally *V. harveyi*; → *Photobacterium damselae* subsp. *piscicida*	[90]
*Vibrio rhodolitus*	Screening and characterization of potential probiotics	_	Isolated from hybrid grouper intestine	Based on simulated gastro-intestinal fluid tolerance, adhesion, digestive enzyme production, antibacterial activity and no disease symptoms or death being shown in grouper, the authors suggested probiotic potential	[86]

N/A—no information available; ↑—increased effect; ↓—decreased effect; →—no effect.

**Table 2:** Effects of promising probiotics on growth performance, immune response, and disease resistance in shellfish and rotifers.

Bacterial Species	Doses	Duration (Days)	Shellfish Species	Parameters Investigated	References
**Gram-positives**					
*Clostridium butyricum*	10^7^ CFU g^−1^	7	Mud crab (*Scylla paramamosain*)	↑ Resistance against *Vibrio parahaemolyticus;* affected the abundance and diversity of microbiota sampled from gut contents of the posterior intestine	[110]
*Microbacterium aquimaris*	In vivo test	_	Isolated from Pacific white shrimp (*Litopenaeus vannamei*) intestine	Revealed N-acyl-homoserine lactone degrading activity; the authors suggested *M. aquimaris* as probiotic candidate for shrimp hatcheries	[111]
*Paenibacillus polymyxa*	10^6^ (PP1), 10^7^ (PP2) and 10^8^ (PP3) CFU g^−1^	60	Pacific white shrimp	↑ Growth, serum, hepatopancreas immune and antioxidant activities, digestive enzyme activities, and intestinal morphology; shaped the gut microbiota composition and disease resistance against *V. parahaemolyticus*	[112]
*P. polymyxa*, *Bacillus coagulans*, and *B. licheniformis*	10^12^ CFU kg^−1^	56	Northern whitings (*Sillago sihama*)	↑ Growth performance and resistance against *V. harveyi*	[113]
**Gram-negatives**					
*Aeromonas media*	10^4^ CFU mL^−1^	5	Pacific oyster (*Crassostrea gigas*)	In vitro studies showed that the strain displayed antagonistic activity towards several shellfish and fish pathogens	[97]
*Enterobacter ludwigii* MA208, *Bacillus amyloliquefaciens* MA228, and *Pediococcus acidilactici* MA160	10^7^ CFU mL^−1^	62	Abalone	↑ Growth performance	[114]
*Phaeobacter*	Doses are presented in the review	Durations are presented in the review	Different shellfish species	The review described health effects and disease resistances	[9]
*Phaeobacter inhibens* DSM 17395	10^7^ CFU mL^−1^	7	European flat oysters (*Ostrea edulis*)	↑ Growth and disease resistance against *V. vulnificus*	[115]
*P. inhibens* S4	10^4^ CFU mL^−1^	7–14	Eastern oyster (*Crassostrea virginica*)	↑ Disease resistance against *Vibrio coralliilyticus;* → growth and survival	[116]
*P. inhibens* S4	10^4^ CFU mL^−1^	7–12	Eastern oyster	Significant effect on bacterial beta-diversity; → effect on alpha-diversity	[117]
*Pseudoalteromonas flavipulchra*	10^8^ CFU mL^−1^	8	Rotifer (*Brachionus plicatilis*)	→ Growth and *Vibrio* counts	[118]
*P. flavipulchra*	10^8^ CFU mL^−1^	8	*Artemia franciscana* nauplii	→ Growth and *Vibrio* counts	[119]
*Pseudoalteromonas piscicida*	Characterisation of a potential new probiotic bacteria	_	*P. piscicida* was isolated from the bivalve (*Modiolus kurilensis*)	The strain showed antimicrobial activity against *Bacillus subtilis*, *Staphylococcus aureus*, and *Candida albicans*, but not against *E. coli* or *P. aeruginosa*	[120]
*P. piscicida* 1UB	10^8^ CFU mL^−1^	40	Pacific white shrimp	↑ Growth performance, immune response, and disease resistance against *V. harveyi*	[121]
*Pseudoalteromonas* sp. F15	10^6^ and 10^6^ CFU mL^−1^	49	Yesso scallop (*Patinopecten yessoensis*)	↑ Specific growth rate; survival; pepsin, amylase and catalase activities; lysozyme, superoxide dismutase and catalase activities; and resistance against *Vibrio splendidus*	[122]
*Pseudoalteromonas*	Characterisation of amylolytic bacteria	_	*Pseudoalteromonas* was isolated from Pacific white shrimp	Revealed high amylolytic content and antimicrobial activity	[123]
A commercial product containing *P. putida*, *L. plantarum*, *L. fermentum*, and *B. subtilis*	N/A	60	Pacific white shrimp	Modulated the bacterial community in water and shrimp intestine	[124]

N/A—no information available; ↑—positive effect; →—no effect.

## Data Availability

No new data were created in this paper as it’s a review of current knowledge.
